# Somatostatin for Bleeding Oesophagitis or Ulceration After Sclerotherapy for Oesophageal Varices

**DOI:** 10.1155/1992/18150

**Published:** 1992

**Authors:** K. E. F. Hobbs

**Affiliations:** University Department of Surgery Royal Free Hospital and School of Medicine NW3 2QG London UK


20. Feu, F., Mas, A., Bosch, J. *et al.* (1988) Domperidone or metoclopramide versus placebo in the prevention of early variceal re-bleeding in cirrhosis. A prospective randomised trial. *J. Hepatology*, **7**, 531
21. Navasa, M., Bosch, J., Rodes, J. (1991) Pharmacological agents and portal hypertension. *In Portal Hypertension*, Ed. Okuda, K., Benhamou, J-P, Springer-Verlag, 35–49

R.A.J. Spence
Consultant Surgeon
Belfast City Hospital
Lisburn Road
Belfast BT9 7AB
Northern Ireland

### SOMATOSTATIN FOR BLEEDING OESOPHAGITIS OR ULCERATION AFTER SCLEROTHERAPY FOR OESOPHAGEAL VARICES

### ABSTRACT

*Jenkins, S.A., Shields, R., Jaser, N., Ellenbogen, S., Makin, C., Naylor, E., Newstead, M., Baxter, J.N. (1991) The Management of gastrointestinal haemorrhage by somatostatin after apparently successful endoscopic injection sclerotherapy for bleeding oesophageal varices. Journal of Hepatology;* **12**: 296–301.

**Twenty-two patients who experienced a severe haemorrhage from either oesophagitis ($n = 8$) or ulcers ($n = 14$) following injection sclerotherapy of their oesophageal varices were treated with intravenous administration of somatostatin (250 μg/h). Somatostatin was effective in controlling haemorrhage and preventing rebleeding in all eight patients bleeding from oesophagitis and in 12 of the 14 patients bleeding from oesophageal ulcers. In two patients with ulcers, haemorrhage persisted despite two periods of concominant balloon tamponade and somatostatin infusion and bleeding was eventually controlled by repeated hourly bolus injections of the hormone for 24 h superimposed on the continuous infusion. The results of this study suggest that somatostatin is an effective and safe treatment for the control of bleeding from either oesophagitis or ulcers following injection sclerotherapy of oesophageal varices.**

### PAPER DISCUSSION

KEY WORDS: Portal hypertension, sclerotherapy, oesophageal ulceration

Mr Jenkins and colleagues have produced the results of another careful study from their Unit on the value of Somatostatin in managing upper gastrointestinal haemorrhage associated with portal hypertension and oesophageal varices. This adds to the existing literature on the value of this therapeutic modality[1]. They have shown previously the value of this drug in the management of acute variceal bleeding and although the exact mechanism of action is unknown, it seemed logical to extend studies into patients suffering from the haemorrhagic complications of injection sclerotherapy.

They found Somatostatin controlled recurrent major haemorrhage after oesophageal bleeding had been arrested initially by injection sclerotherapy. Infusion of the drug successfully treated all their patients who were bleeding from oeosophagitis and most of the patients bleeding from oeosophageal ulcers. For those in whom haemorrhage continued then additional bolus injections were successful.

Apart from pharmacological and injection sclerotherapy techniques, alternative ways of treating acute variceal haemorrhage involve surgical techniques: oesophagogastric disconnection and re-anastomosis using the staple gun with or without gastric devascularisation, acute portal-systemic decompression shunting and liver transplantation. It is unlikely that liver transplantation would be entertained in this situation on a regular basis but acute surgery certainly must. Despite a bad press a few years ago, emergency portacaval shunting, especially small diameter shunts, still has its advocates[2] but injection sclerotherapy is the number one management method in most centres. However it has been shown that if two attempts at controlling bleeding by endoscopic sclerotherapy fail the mortality is unacceptably high. Two independent groups have shown that in this situation a surgical approach must be considered[3,4].

It is probably incorrect for the authors of this paper to say that emergency surgery in these patients carries a "prohibitively high mortality and morbidity". In a carefully controlled prospective study of the results of sclerotherapy compared with oesophageal transection in a group of patients selected for the severity of their disease — they all failed to respond to conservative measures — the mortality and morbidity were comparable. Furthermore the studies showed that later rebleeding was perhaps less frequent and certainly less severe in the transection group than following sclerotherapy[4]. Others have reported transection may offer better results than sclerotherapy especially in lower risk groups[5].

Other studies have shown that rebleeding following staple transection is often due to oesophageal ulceration at the site of the staples[6] which can be readily controlled and treated with Omeprazole[7].

Patients presenting with bleeding oesophageal varices due to liver disease induced portal hypertension must nowadays be considered for eventual liver transplantation. In those who may become candidates then any method of treating the acute problem should not jeopardise this definitive procedure. However the problems associated with carrying out a liver transplant following previous abdominal surgery have probably been overstated. Although the operation may be more taxing to the surgeon there is no recent objective evidence of any increased morbidity or mortality.

Many patients are, of course, not suitable for transplantation and for these it is essential to control the bleeding using a safe technique which will further confer freedom from rebleeding with maximum prolongation of life. It remains to be seen if this will be repeated sclerotherapy, portal decompression — total or selective — or oesophageal transection and devascularisation or pharmacological therapy.

This study clearly demonstrates that the latter concept is viable. However there are cost considerations which must be taken into account. The doses used in this study would cost a British hospital in the order of a thousand pounds for a four day course and additional treatments may be needed. By comparison, Omeprazole costs a little over £10 a week. Surgery is of course more difficult to cost.

This reports an important study with a very positive outcome but it is essential now to investigate further whether the conclusion "that Somatostatin is a safe and effective treatment for post-injection sclerotherapy bleeding from ulcers or oesophagitis" can withstand the rigours of a prospective controlled trial comparing its cost effectiveness with other therapeutic modalities. Clinicians involved in the management of this very difficult group of patients, and I suspect the pharmaceutical industry, will await the results of such studies with great interest.

### REFERENCES

1. Burroughs, A.K. (1991) Somatostatin and Octreotide for Variceal Bleeding. *J. Hepatol.*, **13**(1), 1–4
2. Sarfeh, I.J., Rypins, E.B., Mason, G.R. (1986) A systematic appraisal of portacaval H-Shunt diameters. *Ann. Surg.*, **204**, 356–363
3. Bornman, P.C., Terblanche, J., Kahn, D., Jonker, M.A. and Kirsch, R.E. (1986) Limitations of multiple injection sclerotherapy sessions for acute variceal bleeding. *S. Afr. Med. J.*, **70**, 34–36
4. Burroughs, A.K., Hamilton, G., Phillips, A., Mezzanotte, G., McIntyre, N. and Hobbs, K.E.F. (1989) A comparison of sclerotherapy with staple transection of the esophagus for the emergency control of bleeding from esophageal varices. *New Eng. Med.*, **321**, 857–862
5. Teres, J., Baroni, R., Bordas, J.M., Visa, J., Pera, C. and Rodes, J. (1987) Randomized trial of portacaval shunt, stapling transection and endoscopic sclerotherapy in uncontrolled variceal bleeding. *J. Hepatol.*, **4**, 159–167
6. Kaye, G.L., McCormick, P.A., Sringo, S., Hobbs, K.E.F., McIntyre, N. and Burroughs, A.K. (1991) Staple line erosion: A common source of recurrent bleeding following stapled oesophageal transection. *Br. J. Surg.*, **78**, 1355–57.
7. Kaye, G.L., McCormick, P.A., Sringo, S., Hobbs, K.E.F., McIntyre, N. and Burroughs, A.K. (1992) Bleeding from staple line erosion after esophageal transection: effect of Omeprazole. Hepatology (in press)

K.E.F. Hobbs
Professor of Surgery
University Department of Surgery
Royal Free Hospital and School of Medicine
London NW3 2QG

